# Postoperative extremity gangrene in a patient with type 2 diabetes taking SGLT2 inhibitors: A case report

**DOI:** 10.1097/MD.0000000000025590

**Published:** 2021-04-23

**Authors:** Yun Chin Wong, Kuan-Lin Liu, Chia-Ling Lee

**Affiliations:** a Department of Anesthesiology; b Department of Orthopedics, Hualien Tzu Chi Hospital, Tzu Chi University, Buddhist Tzu Chi Medical Foundation; c Sports Medical Center, Hualien Tzu Chi Hospital, Taiwan.

**Keywords:** case report, euglycemic diabetic ketoacidosis, sodium-glucose cotransporter 2 inhibitors

## Abstract

**Rationale:**

Sodium-glucose cotransporter 2 (SGLT2) inhibitors have been approved and marketed since March 2013. The proportion of patients with type 2 diabetes (T2D) taking SGLT2 inhibitors is increasing. The perioperative adverse effects of SGLT2 inhibitors, especially euglycemic diabetic ketoacidosis (euDKA), should be taken into consideration in perioperative patient evaluation in both elective and emergency surgeries.

**Patient concerns:**

A 57-year-old woman taking SGLT2 inhibitors for T2D developed euDKA after undergoing an emergency orthopedic surgery; the euDKA diagnosis was delayed, thereby causing extremity gangrene.

**Diagnoses:**

EuDKA was diagnosed based on the presence of strongly positive ketonuria, elevated blood beta-hydroxybutyrate level, and severe metabolic acidosis.

**Intervention:**

EuDKA was treated with insulin infusion with dextrose solution and intravenous fluid resuscitation.

**Outcome:**

Due to a delayed diagnosis of euDKA, the patient received a high-dose vasopressor, which led to limb gangrene and amputation 6 months later.

**Lessons:**

EuDKA is often misdiagnosed due to the absence of hyperglycemia. Serum beta-hydroxybutyrate levels or urinalysis could be used as screening tools for euDKA in patients scheduled for emergency surgery, in order to preoperatively administer rapid fluid resuscitation and insulin infusion with dextrose solution, which should continue postoperatively along with serum beta-hydroxybutyrate monitoring.

## 1 Introduction

Sodium-glucose cotransporter 2 (SGLT2) inhibitors represent the newest class of oral antidiabetic drugs approved for type 2 diabetes (T2D) treatment. SGLT2 inhibitors have beneficial cardiovascular and renal effects, and therefore, they have been recommended as second line antidiabetic drugs.^[1]^ Euglycemic diabetic ketoacidosis (euDKA), an adverse effect of SGLT2 inhibitors, is often misdiagnosed, thereby resulting in delayed treatment.^[2]^

## 2 Case presentation

A 57-year-old housewife, who neither drank alcohol nor smoke, had no contributive medical history except T2D evolving for several years, for which she had been taking dapagliflozin/metformin HCL extended release (5/100 mg) tablet for 1 year, with good glycemic control. She was admitted to an orthopedic ward due to left femoral proximal shaft fracture for urgent (open reduction internal fixation) surgery. Preoperative fasting glucose level (115 mg/dl), electrolyte, blood urea nitrogen (14 mg/dl), and creatinine (0.3 ml/dl) levels were within the normal ranges. Her antidiabetic medication was discontinued on the day of surgery, and she was hydrated with dextrose solution. The surgery, which was performed under general anesthesia with nerve block, was uneventful. Intraoperative blood loss and urine output were 400 ml and 200 ml, respectively, and 1200 ml of isotonic crystalloid fluid was infused.

Postoperatively, she experienced neither nausea nor vomiting, and therefore resumed full oral intake of liquids, nutrients, and antidiabetic medication on postoperative day (POD) 1. Although systolic blood pressure on POD 1 intermittently dropped to 85 mm Hg, she had a daily total urine output of 4400 ml and did not complain of any discomfort. On POD 2, she complained of dyspnea and thirst, and presented a deep and rapid breathing pattern. Her spot vital signs were: blood pressure 122/60 mm Hg, heart rate 122/min, respiratory rate 25/min, pulse oxygen saturation 100%, and temperature 36.2°C. Due to the presence of dyspnea, she was immediately administered oxygen using an oxygen mask, and a blood sample was taken for laboratory analysis. Arterial blood gases revealed severe metabolic acidosis with respiratory compensation: pH 6.974, PaCO_2_ 17.8 mm Hg, bicarbonate 4.2 mmol/L, blood glucose 187 g/dl, and lactate 1.1 mmol/L.

She was immediately transferred to an intensive care unit (ICU) and initially treated for septic shock with severe metabolic acidosis and intermittent hypotension. In the ICU, empirical antibiotics, low dose norepinephrine infusion, and isotonic crystalloid hydration were prescribed. The antidiabetic medication was discontinued as the blood glucose level was normal. She was then intubated due to impending respiratory failure and underwent continuous renal replacement therapy due to uncorrectable metabolic acidosis. We performed a series of examinations; nevertheless, the results showed no evidence of a focus of infection. However, urinalysis on POD 3 showed a strongly positive ketonuria (4+), and blood beta-hydroxybutyrate level was 4.5 mmol/L. We had a diagnostic impression of euDKA, and therefore we placed the patient on aggressive hydration with close urine output monitoring.

**Figure 1:**
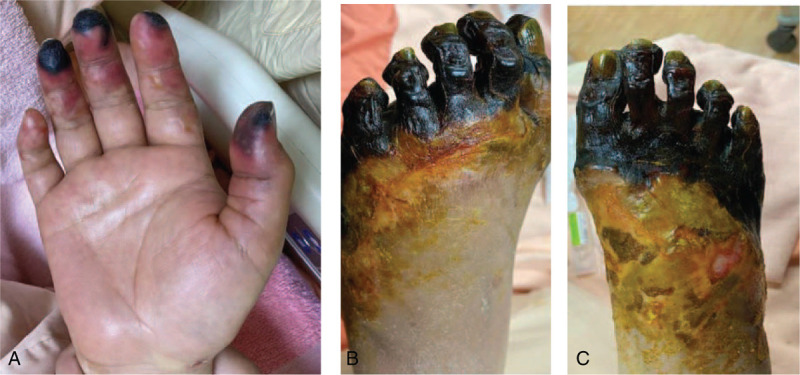
(A), (B), and (C): Dry gangrene of the right finger, right foot, and left foot, respectively, during hospitalization.

Despite a positive fluid balance and renal replacement therapy, ketoacidosis and hypotension persisted; after increasing the dosage of norepinephrine, we noted cyanotic changes on fingers and toes on POD 4. The blood beta-hydroxybutyrate level decreased and metabolic acidosis improved as treatment with intravenous insulin pump was started on POD 6 (Table 1). However, hospital acquired pneumonia with acute respiratory distress syndrome (ARDS) and septic shock developed on POD 14. We increased the dosage of norepinephrine; cyanotic change of extremities aggravated to dry gangrene (Fig. 1). An ARDS protocol was initiated concomitantly with broad spectrum antibiotics. Her condition gradually improved, and therefore she was extubated on POD 45. The dry gangrene of toes and fingers ameliorated as her hemodynamic parameters were stabilized and norepinephrine was discontinued.

**Table 1:** Biochemical analysis results during hospitalization.

	PreOP	POD 1	POD 2	POD 3	POD 4	POD 5	POD 6	POD 7	Range
Na (mmol/L)	138		142	137	144	140	143	142	136–145
K (mmol/L)	3.6		3.4	3.4	3.6	4.1	3.7	3.7	3.5–5.1
GLU (mg/dl)	115	121	158	169	212	155	189	155	70–100
Lactate (mmol/L)			1.1	1.0	1.3	1.1	1.4	1.4	0.5–2.2
pH			6.985	7.35	7.28	7.345	7.327	7.376	7.35–7.45
pCO_2_ (mm Hg)			17.8	20.8	14.7	27.7	40	41	35–45
HCO_3_ (mmol/L)			4.2	11.2	6.8	14.8	22	25.2	22–26
B-ketone (mmol/L)			4.5	6.8	5.6	5.0	5.0	1.5	0–0.6
Urine output (ml)	2400	4400	5100	3090	2510	1670	3420	3181	No range
Fluid input (ml)			3961	7222	5686	4992	4136	4313	No range

**Figure 2:**
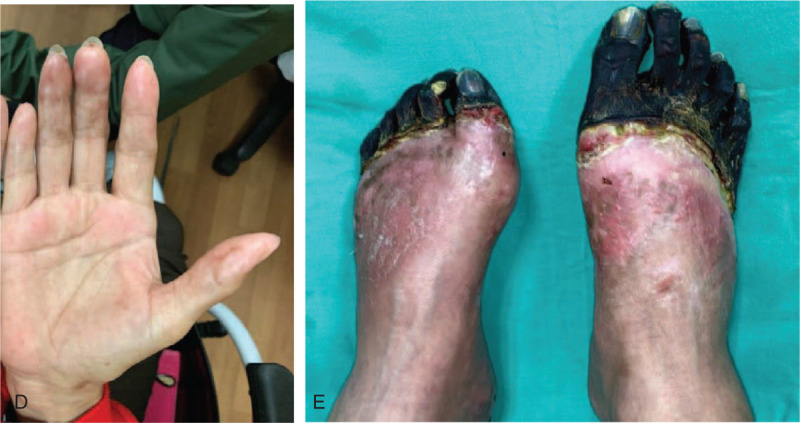
Gangrene of the right fingers (D) showed improvement after 6 months of follow up; however, the toes of both legs with dry gangrene (E) were amputated.

Insulin was discontinued after ketoacidosis reversal; her antidiabetic medication was switched to dipeptidyl peptidase-4 inhibitors, sulfonylureas, and biguanide. The patient was then discharged home from the hospital and followed up at an outpatient clinic. After 6 months of follow up, gangrene of the right fingers showed improvement while toes of both legs with dry gangrene were amputated (Fig. 2).

## 3 Discussion

SGLT2 inhibitors were marketed in 2013 for T2D treatment, and approved as second line oral antidiabetic drugs in Taiwan since 2016. SGLT2 inhibitors act by blocking SGLT2 transporters that are expressed mainly in renal proximal tubules, thereby leading to glycosuria and osmotic diuresis. This mechanism showed an effective lowering of blood glucose level without inducing significant hypoglycemia; further, it reduced systolic blood pressure. SGLT2 inhibitors have been proven to reduce glycated hemoglobin level by 0.7% to 1.0%.^[3]^ Moreover, a recent large randomized controlled trial in patients with T2D showed that SGLT2 inhibitors reduce major adverse cardiovascular events in patients with established atherosclerotic cardiovascular disease, diabetic kidney disease, as well as hospitalization for heart failure.^[4]^ Due to these positive effects, the use of SGLT2 inhibitors is expected to grow rapidly.

However, SGLT2 inhibitors have potential adverse effects, such as an increased risk of genital infection and limb amputation, as well as increased osmotic diuresis and volume depletion. EuDKA is a recognized adverse effect of SGLT2 inhibitors with an incidence rate of 0.1% to 0.3%.^[5]^ EuDKA has similar characteristics as diabetic ketoacidosis, including ketonuria/ketonemia, arterial pH <7.3, serum bicarbonate <15 mEq/L, and an increased anion gap metabolic acidosis. Moreover, patients with euDKA clinically present with tachycardia, hypotension, Kussmaul breathing, dehydration, and euglycemia (blood glucose level <200 mg/dl); the latter is absent in patients with diabetic ketoacidosis.^[6]^ Therefore, euDKA is often misdiagnosed as septic shock during the perioperative period, due to an initial presentation of normal blood glucose level, severe metabolic acidosis, and hypotension. If left undiagnosed, euDKA could deteriorate and cause further complications, such as severe hypovolemia with high dose vasopressor use, which could lead to gangrene.

In our case report, the patient postoperatively developed finger and toe gangrene. Limb amputation had been reported as a potential adverse effect of SGLT2 inhibitors. Does SGLT2 inhibitor-induced euDKA lead to limb gangrene? Currently, there is a lack of strong evidence supporting the association between SGLT2 inhibitors-induced euDKA and limb gangrene; however, the pathophysiology of SGLT2 inhibitor-induced euDKA may provide an explanation. SGLT2 inhibitors cause glycosuria osmotic diuresis, lowering the blood glucose level, which in turn, reduces pancreatic beta cell insulin secretion and triggers pancreatic alpha cell glucagon secretion. Hyperglucagonemia stimulates lipolysis, leading to an increased free fatty acid level and hepatic ketogenesis rate.^[2]^ An ensuing increase in the level of ketone bodies increases blood acidity, thereby causing alkaline and electrolyte derangements. Over time, sodium and potassium depletion ensues, leading to severe dehydration and end organ hypoperfusion, which could be aggravated by an addition of vasoactive agents for hypotension. EuDKA should be treated as DKA with both an adequate intravenous fluid replacement and an insulin infusion with dextrose solution until beta-hydroxybutyrate levels, metabolic acidosis, and bicarbonate levels are normalized.^[7]^

Although perioperative hypo- and hyperglycemia increase complication and mortality rates,^[6]^ SGLT2 inhibitors provide an adequate glycemic control. However, there is no consensus on perioperative oral antidiabetic medication use and dosage, especially pertaining to SGLT2 inhibitors. Most oral antidiabetic medications are recommended for use up to a day before surgery and restarted with normal diet resumption.^[8]^ The half-life of SGLT2 inhibitors ranges from 11 to 13 hours, and the therapeutic effects could persist for days after discontinuation. Due to the potential risk of euDKA development, the Food and Drug Administration recommends SGLT2 inhibitor discontinuation at least 3 days before scheduled surgery.^[9]^

Our patient underwent an urgent orthopedic surgical intervention 24 hours after her last dose of dapagliflozin. She resumed full diet and antidiabetic medication on POD 1 and developed euDKA on POD 2. Diabetes ketoacidosis was not initially diagnosed, and the condition was treated as septic shock using antibiotics and norepinephrine; her extremities were subsequently gangrened. The presence of ketonuria and ketonemia raised our awareness regarding the possibility of SGLT2 inhibitor-associated euDKA, and we administered continuous insulin with dextrose solution and started fluid resuscitation; thereafter, the patient's clinical condition stopped deteriorating. Perioperative fasting, dehydration, blood loss, hypovolemia, and surgical stress may also precipitate euDKA.^[10]^

Hence, we raise an awareness of the possibility of perioperative euDKA development for patients taking SGLT2 inhibitors. SGLT2 inhibitors should be discontinued at least 3 days before scheduled surgery. If emergent surgery is indicated, the patient should be screened for ketonuria and ketosis, and insulin alongside dextrose solution infusion and fluid hydration should be administered until ketoacidosis normalizes. SGLT2 inhibitors should be started upon normal diet resumption. Anesthetists, surgeons, and emergency physicians should raise their index of suspicion of SGLT2 inhibitor-associated euDKA in order to implement an adequate perioperative management.

## Author contributions

**Writing – original draft:** Yun Chin Wong.

**Writing – review & editing:** Kuan-Lin Liu, Chia-Ling Lee.
